# Melanocyte-dependent macrophage redistribution enhances skin immunity upon acute stress

**DOI:** 10.1073/pnas.2511358122

**Published:** 2025-08-19

**Authors:** Erin Faught, Marcel J. M. Schaaf

**Affiliations:** ^a^Department of Animal Sciences, Institute of Biology, Leiden University, Einsteinweg 55, Leiden 2333 CC, The Netherlands

**Keywords:** stress, cortisol, glucocorticoid, immune enhancement, zebrafish

## Abstract

It is well established that stress generally suppresses immunity. However, under certain conditions, acute stress has been shown to stimulate the immune system, particularly those at barrier surfaces like the skin. The cellular and molecular mechanisms underlying this effect remain poorly understood. In the present study, we have identified an immune-enhancing effect of stress using zebrafish larvae. The use of this animal model allowed us to visualize the redistribution of macrophages to the skin upon exposure to an acute stressor, which appeared to be dependent on the increased levels of cortisol. Through real-time imaging of fluorescently labeled leukocytes, we observed that this cortisol-driven redistribution was mediated by both the mineralocorticoid and the glucocorticoid receptor, which upregulated the chemokine receptor Cxcr4. Strikingly, this stress-induced macrophage migration required the presence of melanocytes in the skin, which increased the expression of the gene encoding Cxcl12, the ligand for Cxcr4. This result indicates a pivotal role for pigment cells in immune regulation under stress. Functional assays further revealed that the redistributed macrophages actively increased antigen uptake from the external environment, suggesting an elevated state of immune readiness. Together, we demonstrate that acute stress triggers a coordinated, cortisol-mediated response that enhances immune surveillance at the skin barrier. This stress-induced enhancement of barrier immunity potentially prepares the organism for increased pathogen exposure under challenging conditions.

## Significance

Although stress is often viewed as immunosuppressive, we here reveal a protective effect of acute stress on immunity at barrier surfaces. Using zebrafish larvae, we were able to visualize a cortisol-dependent redistribution of macrophages to the skin. These cells are attracted to the skin by melanocytes, highlighting a unique role for these pigment cells in immune regulation. The redistributed macrophages enhance antigen uptake, which boosts skin immune surveillance, suggesting a conserved stress-adaptive mechanism that may prepare organisms for increased pathogen exposure. These findings advance and refine our understanding of how stress modulates the immune system.

Chronic stress is well known for its ability to suppress immune function. Although the stress response is complex and involves activation of the sympathetic nervous system, alongside the stimulation of the hypothalamus–pituitary–adrenal (HPA) axis, this immune-suppressive effect is primarily mediated by the end result of HPA axis stimulation, the attendant rise in glucocorticoid (GC) hormones, such as cortisol. Based on this, various synthetic GC analogs have clinically been used for decades to treat a wide range of immune-related diseases (1). Aside from the long-term, immune-suppressive effects of GCs, previous studies have provided evidence that short-term stress can instead confer immune protection by boosting the acquisition and expression of immune defenses (1–3). However, little is known regarding the underlying molecular mechanisms and immunological consequences of stress-induced immune enhancement, which precludes any exploitation of this effect to promote human health.

The molecular mechanisms surrounding the immunosuppressive and anti-inflammatory actions of endogenous GC hormones and synthetic GC drugs are well characterized. Most of these effects are attributable to the transcriptional activation of the glucocorticoid receptor (GR). This receptor is one of two receptors for cortisol, the other being the high-affinity mineralocorticoid receptor (MR), which does not appear to contribute appreciably to the anti-inflammatory effect of GCs. GR will act to suppress the transcription of immune-stimulatory genes such as genes encoding proinflammatory chemokines and cytokines (e.g., TNF-α, IL-8, IL-6), largely by interacting with other transcription factors, like AP-1 or NF-kB, thereby inhibiting their action (2, 4). In addition, GR can increase transcription of anti-inflammatory genes (e.g., GILZ, IκBα, IL-10) by binding to glucocorticoid response elements (GREs) in the DNA and recruiting specific sets of transcriptional coregulators (4). Finally, GR can also mediate rapid effects, altering the metabolism of immune cells, thereby driving potent anti-inflammatory macrophage phenotypes (5).

In addition to these well-established immune-suppressive effects, it has also been demonstrated that GC exposure can enhance immune responses. Whereas cytokine levels are often suppressed by GCs, the expression of various receptors for these cytokines (e.g., IL-1, IL-2, IL-4, IL-6, and IFNγ) has been shown to be upregulated by GCs in various immune cell types, effectively resulting in an accelerated immune response (6). In addition, in cells of the innate immune system, the expression of complement factors, scavenger receptors, and several pattern-recognition receptors (PRRs), such as TLR2, NLRP3, and P2Y2R, has been shown to be upregulated by GCs (1, 7–9). These responses are thought to involve GR, but few studies have explicitly explored this, and to date, there is no evidence of the involvement of MR. The immune-enhancing effects of GCs appear to be largely dependent on both duration and dosage (2). For example, while low-dose corticosterone administration significantly enhanced a delayed-type hypersensitivity (DTH) response in mice (10) and increased proinflammatory gene expression in mouse peritoneal macrophages (11), high-dose corticosterone significantly suppressed these responses. This is in line with the working hypothesis that long-term stress is immune-suppressive, and short-term stress/GC treatment promotes the immune response (12).

The immunological consequences of stress-induced immune enhancement are also unclear. However, a commonly observed phenotype associated with stress is the redistribution of leukocytes. Changes in blood leukocyte numbers poststress are an evolutionarily conserved response that has been observed in fish [killifish (13)], rodents (14–16), nonhuman primates (17), and humans (18–21), supporting the notion that redistribution of immune cells poststress is functionally significant. Subsequent studies have demonstrated that upon acute stress, leukocytes are transported through the blood to peripheral sites as the skin, the mucosal lining of gastrointestinal and urinary-genital tracts, the lung, the liver, and the lymph nodes, enhancing local immunity at these sites (3). Mechanistically, the DTH reaction has been used to model skin-cell mediated immune responses, where stress-induced enhancement of the DTH response was shown to be dependent on GC hormones, such as cortisol (corticosterone in rodents), and on interferon-γ (IFN-γ) signaling (22). Moreover, it was shown that this immune-enhancing effect of stress was only observed upon acute stress or corticosterone administration, whereas chronic stress or corticosterone treatment suppressed the DTH response (12). However, little is known about the mechanisms underlying the GC-induced redistribution of leukocytes to the skin upon acute stress.

In the present study, we aimed to uncover the molecular mechanisms that are associated with innate immune cell redistribution poststress and whether this is part of a wider paradigm of immune protection. For this purpose, we have used the zebrafish as our animal model. The innate immune system of the zebrafish develops within a few days postfertilization (dpf), whereas the adaptive immune system only matures after two weeks, which means the innate immune system can be studied separately in zebrafish larvae (23, 24). The development of the zebrafish innate immune system shows close correspondence to the development of its mammalian equivalent, and myelopoiesis is regulated by conserved molecular pathways. As in mammals, myelopoiesis in zebrafish occurs in successive waves (reviewed in ref. 25). Primitive myelopoiesis begins with myeloid specification in the anterior lateral plate mesoderm, leading to macrophage and neutrophil populations that dominate by 2 dpf. Later, definitive myelopoiesis arises transiently in the caudal hematopoietic tissue (CHT) and is ultimately established in the kidney. By 3 dpf, these definitive neutrophils and macrophages largely replace the primitive leukocyte populations. Based on their origin, form, and function, zebrafish neutrophils and macrophages are generally considered to be the cellular orthologs of their mammalian counterparts. Importantly, since zebrafish larvae are optically transparent, transgenic lines with fluorescently labeled macrophages (26, 27) and neutrophils (28) enable the visualization of the location and behavior of leukocytes. In several previous studies, we have shown that this model is highly suitable to investigate the interaction between stress, GCs, and the innate immune system (29, 30).

Here, we show that macrophages are localized quickly toward the skin upon acute stress and that this phenotype persists for at least 24 h. We further establish that the melanocytes in this region orchestrate this stress-induced redistribution and that they do so by increasing activity of the Cxcr4/Cxcl12 signaling axis. Finally, we observe that these stress-activated macrophages show increased uptake of soluble antigens, supporting the notion that acute stress promotes skin immunity through this process.

## Results

### Leukocytes Rapidly Redistribute to the Dorsal Periphery Following Acute Stress.

**Fig. 1.**
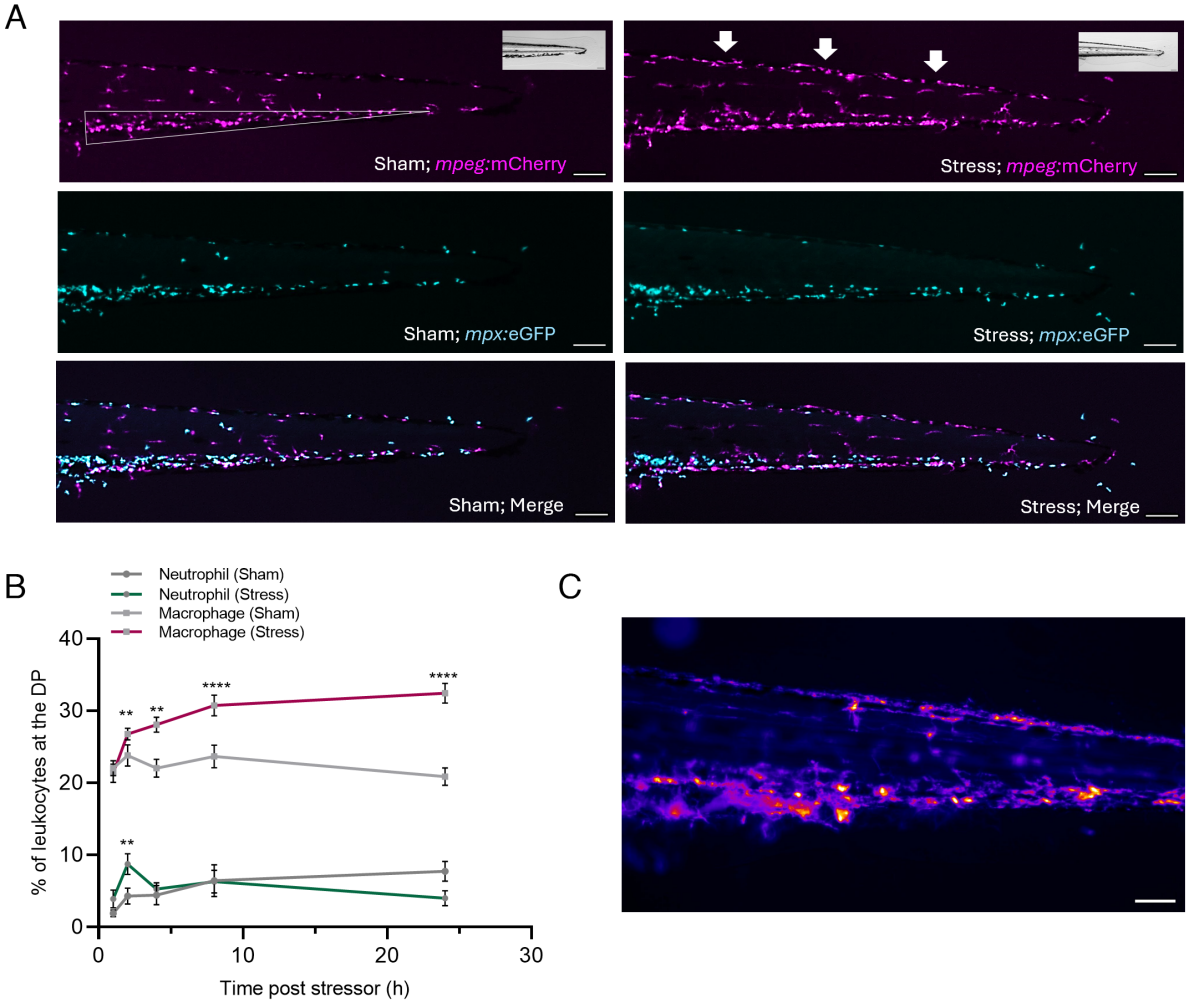
Acute stress causes redistribution of leukocytes toward the DP. (*A*) Representative images of the posterior tail region of *Tg(mpeg:mCherry/mpx:eGFP)* larvae showing the location of macrophages (*mpeg*+; magenta) and neutrophils (*mpx*+; cyan), 24 h postacute stress. The white triangle denotes the caudal hemopoietic tissue (CHT), and the arrows denote the DP. (Scale bar, 100 µm.) (*B*) Percentage of macrophages (*mpeg*+) and neutrophils (*mpx*+) that migrate toward the DP at 1, 2, 4, 8, and 24 h postacute stress in *Tg(mpeg:mCherry/mpx:eGFP)* larvae. Data shown represent mean ± SEM. **P < 0.01, ****P < 0.0001. (*C*) Representative heat map image of select macrophages over the first 24 h poststress in *Tg(mpeg:mCherry/mpx:eGFP)* larvae. The heat map was generated from the mpeg:mCherry channel of Movie S1.

**Fig. 2.**
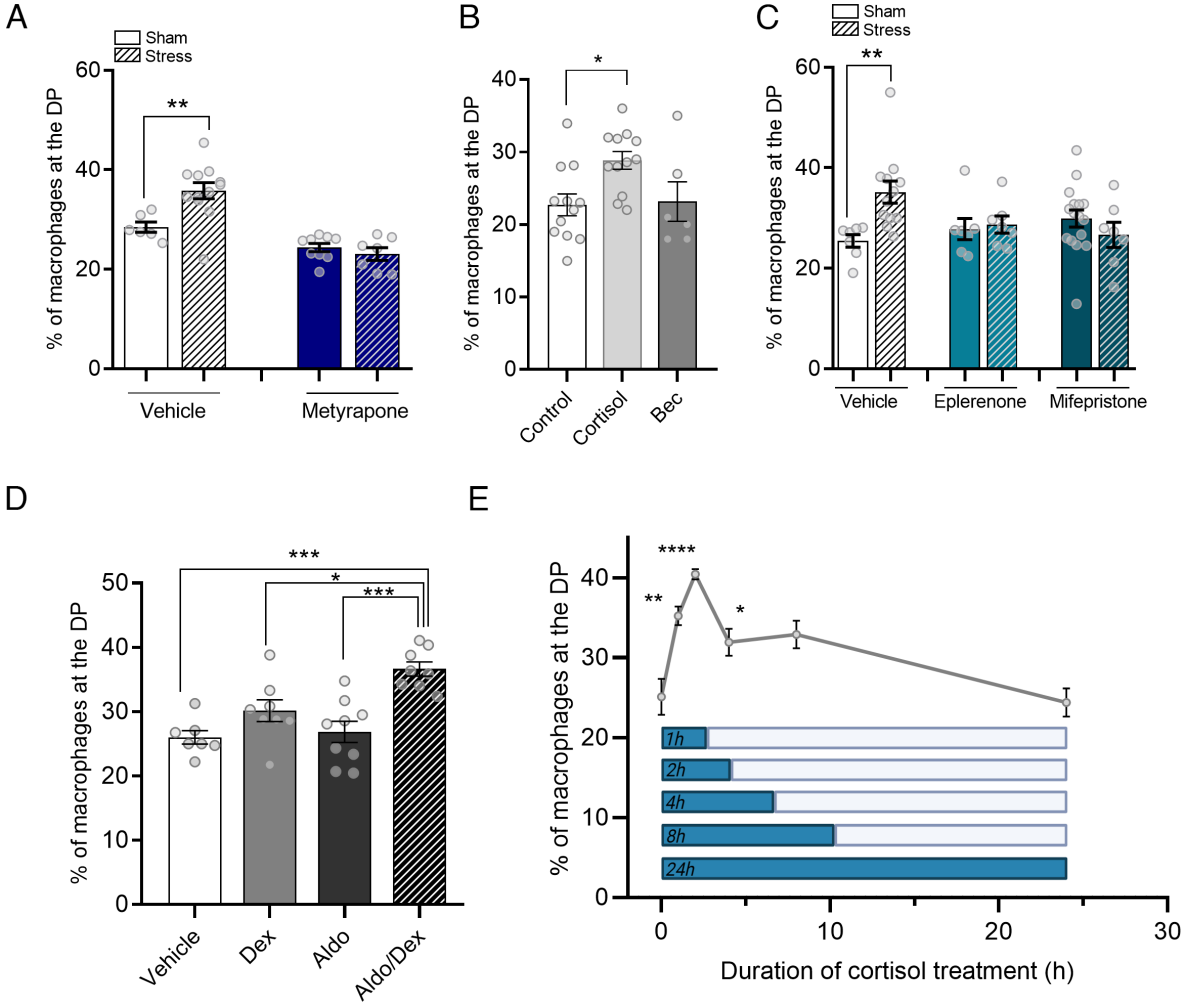
Stress-induced macrophage redistribution is dependent on cortisol signaling through MR and GR. (*A*) Percentage of macrophages that redistribute to the DP 24 h postacute stress in *Tg(mpeg:mCherry)* larvae, treated with either vehicle (0.05% ethanol) or metyrapone (100 µM; 16 h). (*B*) Percentage of macrophages (*mpeg*+) that migrate toward the DP 24 h postacute glucocorticoid treatment in *Tg(mpeg:mCherry)* larvae, treated with either vehicle (0.05% ethanol), cortisol (hydrocortisone; 25 µM; 1 h), or beclomethasone (Bec; 25 µM; 1 h). (*C*) Percentage of macrophages that migrate toward the DP postacute stress in *Tg(mpeg:mCherry)* larvae, treated with either vehicle (0.05% ethanol), eplerenone (MR antagonist; 1.25 µM; 16 h), or mifepristone (GR antagonist; 1.25 µM; 16 h). (*D*) Percentage of macrophages (*mpeg*+) that migrate toward the DP 24 h postacute glucocorticoid/mineralocorticoid treatment (1 h) in *Tg(mpeg:mCherry)* larvae, treated with either vehicle (0.1% ethanol), dexamethasone (Dex; 10 µM; 1 h), aldosterone (Aldo; 10 µM; 1 h), or the combined treatment (Aldo/Dex). (*E*) Percentage of macrophages that migrate toward the DP in *Tg(mpeg:mCherry)* larvae after exposure to cortisol (25 µM) for 0, 1, 2, 4, 8, and 24 h. The inset depicts a schematic overview of the cortisol duration experiment. Quantification was done 24 h after the start of the cortisol exposure. In all graphs, the data shown represent mean ± SEM, and the dots represent single larvae. *P < 0.05, **P < 0.01, ****P < 0.0001.

To evaluate whether acute stress resulted in altered leukocyte distribution and function, we subjected larvae (3 dpf) to an acute swirling stressor and examined the behavior of neutrophils and macrophages over a period of 24 h. Under resting conditions, both macrophages and neutrophils primarily populate the CHT (Fig. 1*A*), with a small subset of each leukocyte type being present throughout the tail region and along the dorsal periphery (DP). In the CHT, the numbers of macrophages and neutrophils are approximately equal (31). However, the percentage of macrophages that are present along the DP of the tail region (22.1 ± 1.1% at 0.5 h in sham-treated larvae; expressed as a percentage of the total number of macrophages present in the tail; Fig. 1*B*) was nearly 10-fold higher compared to the percentage of neutrophils (2.6 ± 0.5%; Fig. 1*B*), suggesting cell-specific roles. Postacute stress, both macrophages and neutrophils show increased migration toward the DP. The percentage of macrophages localized in the DP increases by 2 h (26.3 ± 1.2%; *P* = 0.008) and remains elevated at 4 h (28.1 ± 1.0%; *P* = 0.0013), 8 h (30.7 ± 1.5%; *P* < 0.0001), and 24 h (32.5 ± 1.4%; *P* < 0.0001; Fig. 1*B*) postacute stress, in comparison to the 1 h poststress (hps) time point (21.3 ± 1.3%). Of note, while acute stress rapidly increased the percentage of patrolling macrophages by 2 hps, there was a further increase by 24 hps (2 hps vs. 24 hps: *P* = 0.008). Because we found maximal macrophage redistribution at 24 hps, we focused on this latter time point in the remainder of this work. A similar response was observed for the neutrophils. Their number rapidly increased threefold by 2 hps in this area (8.7 ± 1.4%; *P* = 0.005; Fig. 1*B*). However, this increase is very transient (already absent at 4 hps) and is still less than half the number of macrophages already patrolling the area, suggesting that neutrophils may play a minimal or brief role in poststress immune enhancement. While the previous figures show a sequential line of macrophages along the DP, further live imaging showed that the macrophages will patrol this area, moving alongside and around the dorsal longitudinal anastomosing vessel (DLAV) (Movie S1 and Fig. 2*C*). A heat map of macrophage movement from 2 to 24 h poststress clearly shows that while macrophages in the CHT are relatively static, macrophages localized to the DP will cause a more evenly distributed track over time (Fig. 1*C*).

We subsequently studied whether this leukocyte redistribution was stressor-specific. Previous work has established that, at this point in larval development, an osmotic challenge was an effective stressor (32, 33). Therefore, we subjected larvae to either 500 mM NaCl, or 250 mM NaCl for 5 min and allowed recovery for 24 h. Similar to the response to swirling stress, the number of macrophages along the DP nearly doubled after this stressor (sham: 18.3 ± 1.2% vs. full-strength seawater: 26.1 ± 5.3%; *P* = 0.039; *SI Appendix*, Fig. S1*A*), demonstrating that macrophage redistribution is not stressor-specific.

Distinct from the stressor-induced response, we observed that neutrophils also showed increased migration along the DP at 24 hps in response to 500 mM NaCl (sham: 9.8 ± 3.4% vs. 500 mM NaCl: 31.0 ± 8.0%; *P* = 0.0376; *SI Appendix*, Fig. S1*B*). This suggests that either the osmotic challenge may be a more effective stressor and therefore able to mobilize neutrophils more effectively, or that an osmotic challenge elicits a different response, for example, by causing damage to the skin which may induce a neutrophilic inflammatory response (*SI Appendix*, Fig. S1*C*).

Since both swirling and osmotic stress could potentially cause damage to the skin, we also used light as a stressor to eliminate tissue damage as a confounding factor. In the light stress procedure (34), larvae were acclimated for 15 min in the dark, followed by 10 min in the light. This resulted in a 2-fold increase in cortisol levels (sham: 7.4 ± 0.6 pg/larva vs. stressed: 15.8 ± 2.6 pg/larva; *P* = 0.0141; *SI Appendix*, Fig. S1*D*). Light stress was, similar to the other two stressors, also associated with an increase in the number of macrophages that were localized at the DP (sham: 23.9 ± 2.1% vs. stressed: 30.8 ± 1.1%; *P* = 0.0083; *SI Appendix*, Fig. S1 *E* and *F*). Taken together, these data show that the redistribution of macrophages poststress appears to be stressor-independent.

### Acute Stress–Induced Macrophage Redistribution Is Dependent on Cortisol Signaling.

To test whether the redistribution of macrophages poststress was due to the attendant rise in cortisol, and not a suite of other hormones released during stress, we treated larvae, at 16 h prior to the swirling stress, with the cortisol biosynthesis inhibitor metyrapone. This treatment, indeed, abolished the stress-induced increase in cortisol levels (*SI Appendix*, Fig. S2*A*). Accordingly, while acute stress increased the percentage of macrophages that migrated toward the DP in vehicle-treated larvae (sham: 28.5 ± 1.0% vs. stressed: 35.8 ± 1.6%; *P* = 0.083), this effect was abolished in metyrapone-treated larvae (Fig. 2*A*). This indicates that the increase in cortisol levels is a key step in the mechanism underlying stress-induced macrophage redistribution.

Indeed, treatment with cortisol (1 h), appeared to be sufficient to increase macrophage redistribution (*P* = 0.043; Fig. 2*B*). To determine which corticosteroid receptor mediated the cortisol-induced macrophage redistribution, we next treated larvae with several GR agonists (1 h). Unlike cortisol, beclomethasone did not alter macrophage redistribution (Fig. 2*B*), nor did prednisolone or dexamethasone (*SI Appendix*, Fig. S2*B*). These results indicate that activation of GR alone is not sufficient and that likely MR activation is required as well. To study this, we next treated larvae with eplerenone (an MR antagonist) and mifepristone (a GR antagonist), 16 h prior to the stressor. Treatment with either antagonist abolished the migratory macrophage response to stress, while in vehicle-treated larvae the percentage of macrophages that migrated to the DP was increased upon stress (sham: 25.4 ± 1.2% vs. stressed: 35.1 ± 2.2%; *P* = 0.0055; Fig. 2*C*). We further confirmed the dependency of macrophage redistribution on MR and GR by blocking the effect of acute cortisol treatment with eplerenone and mifepristone (*SI Appendix*, Fig. S2*C*). Additionally, whereas individual treatment with dexamethasone (GR agonist) or aldosterone (MR agonist) had no effect on macrophage redistribution, cotreatment with both agonists was able to induce macrophage redistribution (Fig. 2*D*). These data indicate that both MR and GR are involved in mediating the effects on macrophage distribution of cortisol poststress.

Having established that cortisol mediates the redistribution of macrophages after acute stress, we were interested in whether this effect was dependent on the duration of the exposure to cortisol. To test this, we treated fish with cortisol for 1, 2, 4, 8, and 24 h and determined the macrophage distribution at 24 h after the start of these treatments (Fig. 2 *D*, *Inset*). While treatment of cortisol for 1 h rapidly increased macrophage redistribution toward the DP (35.2 ± 1.2%), compared to the sham larvae (25.1 ± 2.3%; *P* = 0.0046), we observed that the peak in macrophage redistribution occurred when larvae were treated with cortisol for 2 h (40.5 ± 0.6%; *P* < 0.0001). Longer cortisol treatment, at 4 h (31.9 ± 1.7%; *P* = 0.0637) and 8 h (32.9 ± 1.8%; *P* < 0.0260) only showed a modest increase compared to the sham larvae. Treatment with cortisol for the full 24 h recovery period resulted in no effect on macrophage redistribution (Fig. 2*D*), suggesting that long-term cortisol treatment has a distinct phenotype from short-term treatment.

### Cxcr4a Is a Key Regulator of Acute Stress–Induced Macrophage Redistribution.

**Fig. 3.**
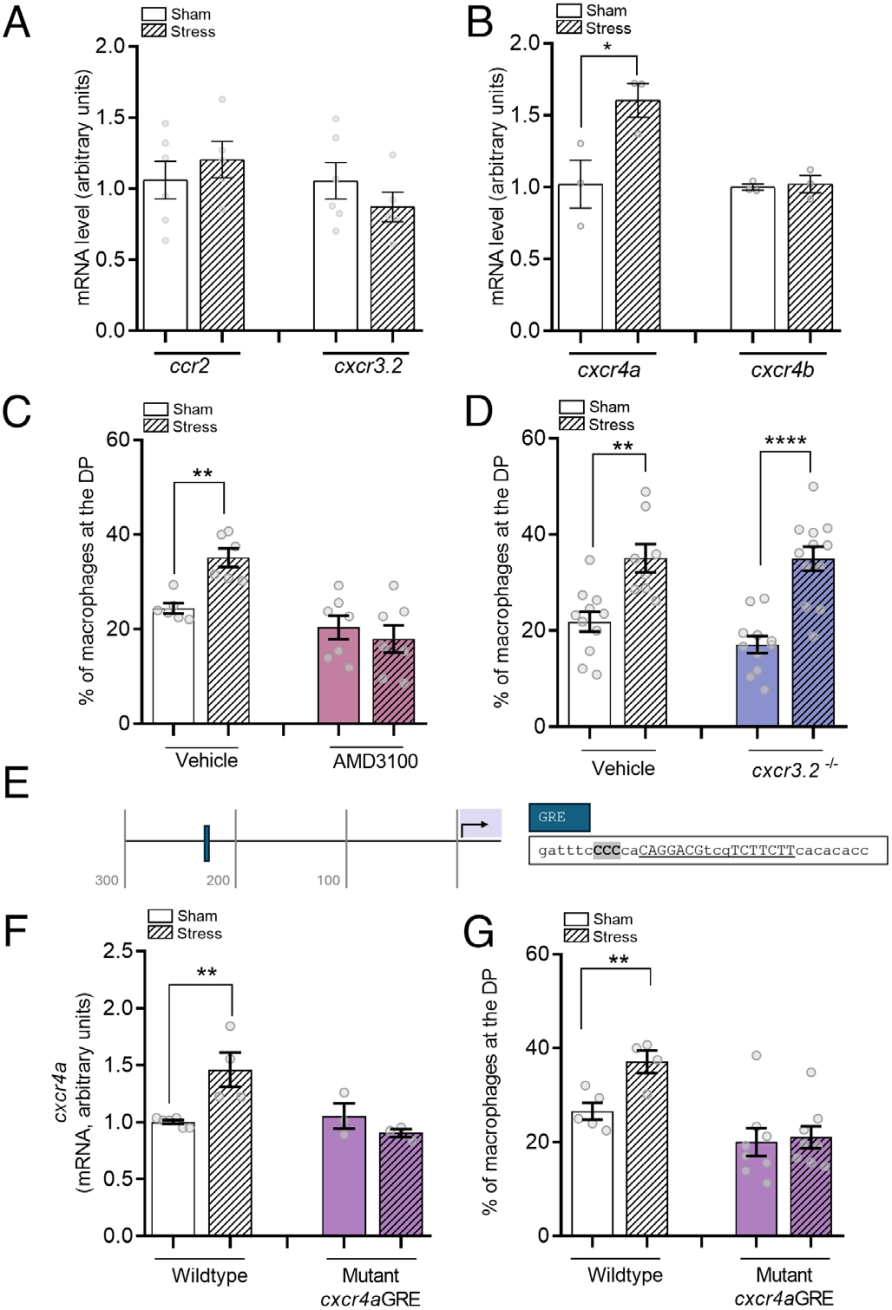
Cxcr4a is a key regulator of acute stress–induced macrophage redistribution. (*A*) Transcript abundance of genes encoding chemokine receptors, *ccr2* and *cxcr3.2*, in sham and stressed larvae. (*B*) Transcript abundance of genes encoding chemokine receptors, *cxcr4a* and *cxcr4b*, in sham and stressed larvae. (*C*) Percentage of macrophages that migrate toward the DP 24 h postacute stress in *Tg(mpeg:mCherry)* larvae treated with either vehicle or the Cxcr4 antagonist AMD3100. (*D*) Percentage of macrophages that migrate toward the DP postacute stress in *Tg(mpeg:mCherry);cxcr3.2^+/+^ or Tg(mpeg:mCherry);cxcr3.2^−/−^* larvae. (*E*) Schematic diagram showing the proximal promoter of the *cxcr4a* gene. The protospacer motif (PAM) site is highlighted in gray. The putative GRE site is shown in capital letters. The arrow shows the transcriptional start site. (*F*) Transcript abundance of *cxcr4a* in wild-type larvae, or crispant larvae with a disruption of a GRE in the promoter of *cxcr4a*. (*G*) Percentage of macrophages that migrate toward the DP 24 h postacute stress in wild-type larvae, or crispant larvae with a disruption of a GRE in the promoter of *cxcr4a*. In all graphs, data shown represent mean ± SEM, and dots represent individual measurements (pools of 10 larvae in *A*, *B*, and *F*, single larvae in *C*, *D*, and *G*). *P < 0.05, **P < 0.01, ****P < 0.0001.

While we established that cortisol signaling was necessary to mobilize macrophages toward the DP, we next sought to elucidate the underlying molecular mechanism(s). Chemotaxis of macrophages and the associated molecular mechanisms are well characterized in zebrafish. Here, we examined the expression of genes encoding receptors known to be involved in macrophage migration (Ccr2, Cxcr3.2, and Cxcr4) (30, 35, 36). Our results showed that acute stress did not affect the transcript abundance of *ccr2* and *cxcr3.2* (Fig. 3*A*). However, there was an increase in *cxcr4a* expression (Fig. 3*B*; *P* = 0.0456), suggesting that this receptor may be a key modulator of stress-induced macrophage chemotaxis. The expression of the second *cxcr4* paralogue gene in zebrafish, *cxcr4b*, was not altered upon stress (Fig. 3*B*).

To further test the involvement of the Cxcr4 receptor, we used the Cxcr4 antagonist AMD3100, which had previously been validated in zebrafish (37). Treatment of the larvae with AMD3100 abolished the increase in the percentage of macrophages present at the DP that was observed in vehicle-treated larvae (sham: 24.4 ± 1.1% vs. stressed: 35.1 ± 1.9%; *P* = 0.0008; Fig. 3*C*). These data confirm that Cxcr4 is a key player in modulating stress-induced macrophage redistribution. Subsequently, we tested whether another key regulator of macrophage migration, Cxcr3.2, was involved. Here, we used transgenic *cxcr3.2* knockout larvae (*cxcr3.2^−/−^*) that contained fluorescently labeled macrophages and neutrophils (*Tg(mpeg:mCherry/mpx:eGFP*)). While acute stress nearly doubled the percentage of macrophages at the DP (sham: 21.8 ± 2.9% vs. stressed: 35.1 ± 2.9%; *P* = 0.0015; Fig. 3*D*), a similar result was observed in the *cxcr3.2* knockout larvae (*P* < 0.0001). This again suggests that acute stress acts specifically through Cxcr4 to redistribute macrophages toward the DP, via upregulation of the *cxcr4a* paralogue.

However, whether MR and GR directly modulate the increase in *cxcr4a* expression was still unclear. Therefore, we next disrupted a putative GRE in the *cxcr4a* gene, using CRISPR/Cas9 (Fig. 3*E*). Guide RNA was designed to target a predicted GRE site (position: chr6:12921929-12921945; *P* > 0.001; caggacgtcgtcttctt; Fig. 3*E*) and was determined using the JASPAR TFBS2022 track of the University of Santa Cruz (UCSC) genome browser (genome build: GRCz11/danRer11). The GRE site was located 243 bp upstream of the transcriptional start site, and 3 bp downstream of a Cas9 PAM site. We first established that the site was effectively mutated by examining a shift in melt curve temperature as a readout of amplicon size (*SI Appendix*, Fig. S3*C*). Next, we confirmed that there were minimal off-target effects on other GREs. To do this, we treated larvae with cortisol for 6 h, which was sufficient to increase *fkbp5* transcript abundance 25-fold (*P* < 0.0001; *SI Appendix*, Fig. S3*D*), and this induction was unaffected in the *cxcr4a* GRE crispant larvae, indicating that GREs in the *fkbp5* gene were not targeted. Interestingly, in larvae harboring a disruption in the *cxr4a* GRE site, acute stress did not result in an alteration in *cxcr4a* mRNA levels, whereas a 40% increase was observed in wild-type larvae (*P* = 0.005; Fig. 3*F*). Finally, we examined whether this GRE disruption would also alter the stress-induced macrophage redistribution. In the larvae with a disrupted *cxcr4a* GRE, the percentage of macrophages in the DP remains unchanged upon stress, while it increased in the wild-type larvae (sham: 26.6 ± 1.8% vs. stressed: 37.1 ± 2.4%; *P* = 0.0089; Fig. 3*G*). Taken together, our data suggest that not only is the upregulation of *cxcr4* expression a key mechanistic step in the stress-induced macrophage redistribution but also that the GRE in the proximal promoter is critical for this induction, likely due to binding of both GR and MR.

### Macrophage Redistribution Poststress Is Driven by Melanocyte-Derived Cxcl12a.

**Fig. 4.**
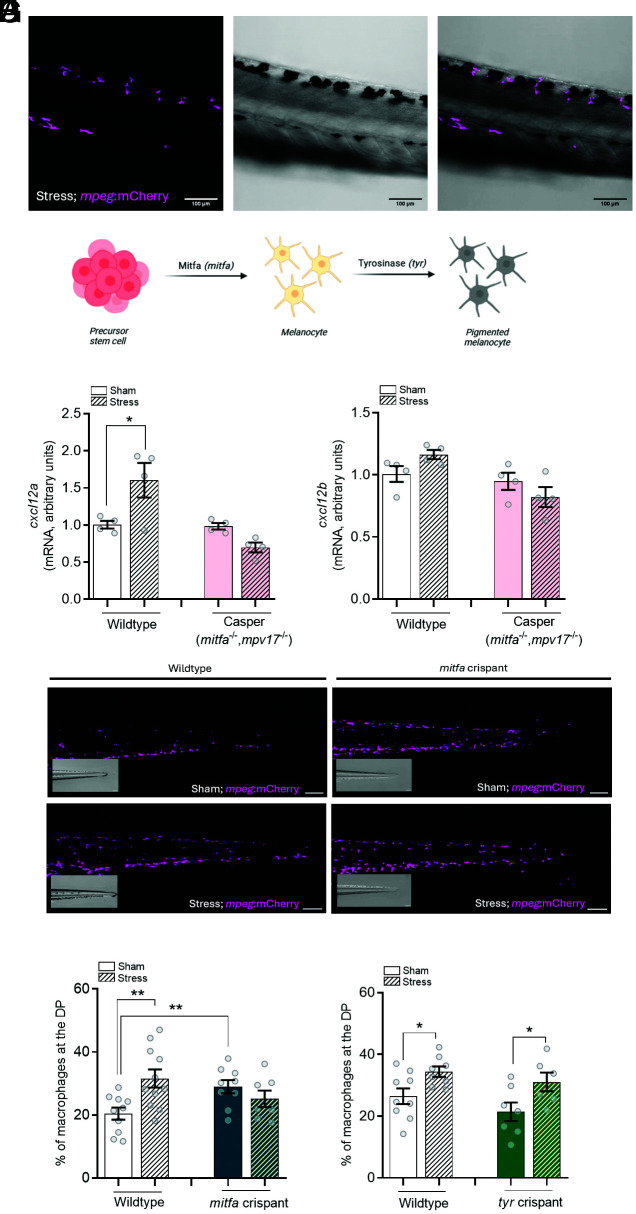
Macrophage redistribution is driven by melanocyte-derived Cxcl12a. (*A*) Representative fluorescence and brightfield microscopy images of the DP of *Tg(mpeg:mCherry)* larvae that show localization of macrophages (*mpeg*+; magenta) in close vicinity of melanocytes in the DP. (Scale bar, 100 µm.) (*B*) Schematic outline of melanocyte differentiation, depicting the roles of Melanocyte-inducing transcription factor a, a transcription factor involved in melanocyte maturation (encoded by the *mitfa* gene), and Tyrosinase, an enzyme involved in pigmentation (encoded by the *tyr* gene). (*C*) Transcript abundance of *cxcl12a* in sham and stressed wild-type and Casper (*mitfa^−/−^;mpv17^−/−^*) larvae. Bars represent mean ± SEM. Each value represents a pool of 10 larvae. (*D*) Transcript abundance of *cxcl12b* in sham and stressed wild-type and Casper (*mitfa*^−/−^*;mpv17*^−/−^) larvae. (*E*) Representative images of the tail region of wild-type *Tg(mpeg:mCherry)* larvae and *mitfa* crispant *Tg(mpeg:mCherry)* larvae. (Scale bar, 100 µm.) (*F*) Percentage of macrophages that migrate toward the DP in wild-type or *mitfa* crispant larvae subjected to stress. (*G*) Percentage of macrophages that migrate toward the DP in wild-type or *tyr* crispant larvae subjected to stress. In all graphs, data shown represent mean ± SEM and dots represent individual measurements (pools of 10 larvae in *A* and *B*, single larvae in *F* and *G*). *P < 0.05, **P < 0.01.

We next asked what drives the Cxcr4-based chemotaxis of the macrophages to the DP poststress. In particular, we found the distribution of the macrophages poststress to be distinctive. selectively redistributing toward areas of melanocyte development (Fig. 4*A*), which also develop along the DP, in addition to other areas around the body (38). Therefore, we postulated that macrophages migrate toward the melanocytes in response to the release of a chemoattractant by these cells due to the release of the Cxcr4 ligand, Cxcl12. To determine whether melanocytes played a role, we used the Casper mutant zebrafish line, which has a complete lack of melanocytes, due to a mutation in the gene encoding melanocyte-inducing transcription factor a (*mitfa/nacre*), as well as iridophores (due to a loss of *mpv17/roy* gene). Mitfa is a key regulator of melanocyte development, which is required at multiple stages of melanocyte differentiation, whereas pigmentation by melanin is due to the action of the oxidase enzyme Tyrosinase (encoded by the *tyr* gene) (Fig. 4*B*) To test the hypothesis that the levels of a Cxcl12 paralogue were regulated by melanocytes, we stressed Casper larvae and measured the transcript abundance of *cxcl12a* and *cxcl12b*. The *cxcl12a* mRNA levels increased ~40% (*P* = 0.0463) in the whole body upon stress, and this was abolished in Casper larvae (Fig. 4*C*). There was no change in the *cxcl12b* transcript levels poststress (Fig. 4*D*). These data suggest that melanocyte-derived Cxcl12 levels attract the macrophages to the DP poststress.

Because the Casper fish lack both melanocytes and iridophores, we next wanted to confirm that it was the melanocytes, specifically, that were responsible for driving the stress-induced increase in *cxcl12a* expression. To accomplish this, we created *mitfa* crispants in *Tg(mpeg:mCherry/mpx:eGFP)* larvae and quantified the leukocyte distribution postacute stress. Disruption of *mitfa* indeed resulted in a loss of melanocytes (Fig. 4 *E*, *Inset*), and the effect of stress on macrophage migration to the DP was abolished in the *mitfa* crispants (Fig. 4 *E* and *F*). This may mean that we have lost the stress phenotype in the *mitfa* crispants because the number of macrophages at the DP is already at a maximal level. Finally, disruption of the *tyr* gene did not alter acute stress–induced macrophage redistribution (Fig. 4*G*), indicating that, while melanocytes require melanin for proper functioning, the tyrosinase-mediated production of melanin is not required for this specific effect. Overall, our data point to an important role for melanocytes in the distribution pattern of innate immune cells in response to acute stress.

### Acute Stress Promotes Barrier Immunity.

Having established how macrophages reach the periphery, we next wanted to investigate the immunological significance of this migration. We postulated that the reason macrophages migrate to the periphery poststress would be to increase barrier immunity as part of a stress-induced immune enhancement program. Using another marker of macrophages, microfibril-associated protein 4 (*mfap4*) in a double transgenic line (*Tg(mpeg:eGFP/mfap4:mCherry*)), we confirmed that while *mpeg*-positive cells increased their migration toward the DP after stress (sham: 21.0 ± 2.9% vs. stressed: 35.2 ± 4.3%; *P* = 0.0475; *SI Appendix*, Fig. S5*A*), there was no effect of stress on the *mfap4*-positive cell distribution (sham: 20.8 ± 5.4% vs. stressed: 19.9 ± 3.0%; *SI Appendix*, Fig. S5*A*). This suggests that the *mpeg*-positive cells that are stress-responsive are a subset of macrophages, which are *mfap4*-negative. The presence of *mpeg*-positive and *mfap4*-negative macrophage-like cells in the periphery has previously been shown in zebrafish, and these macrophage-like cells were further characterized to be metaphocytes, capable sampling soluble antigens using transepithelial protrusions (39). In our study, we were unable to confirm whether the redistributed subset of macrophages were specifically metaphocytes, because exposure to acute stress had no, or only a modest, effect on several metaphocyte markers (*SI Appendix*, Fig. S5*C*). The fact that the redistributed cells are mfap4^-^ also eliminates Langerhans cells as a possible cell type, as these cells also express *mfap4* in zebrafish (40, 41).

**Fig. 5.**
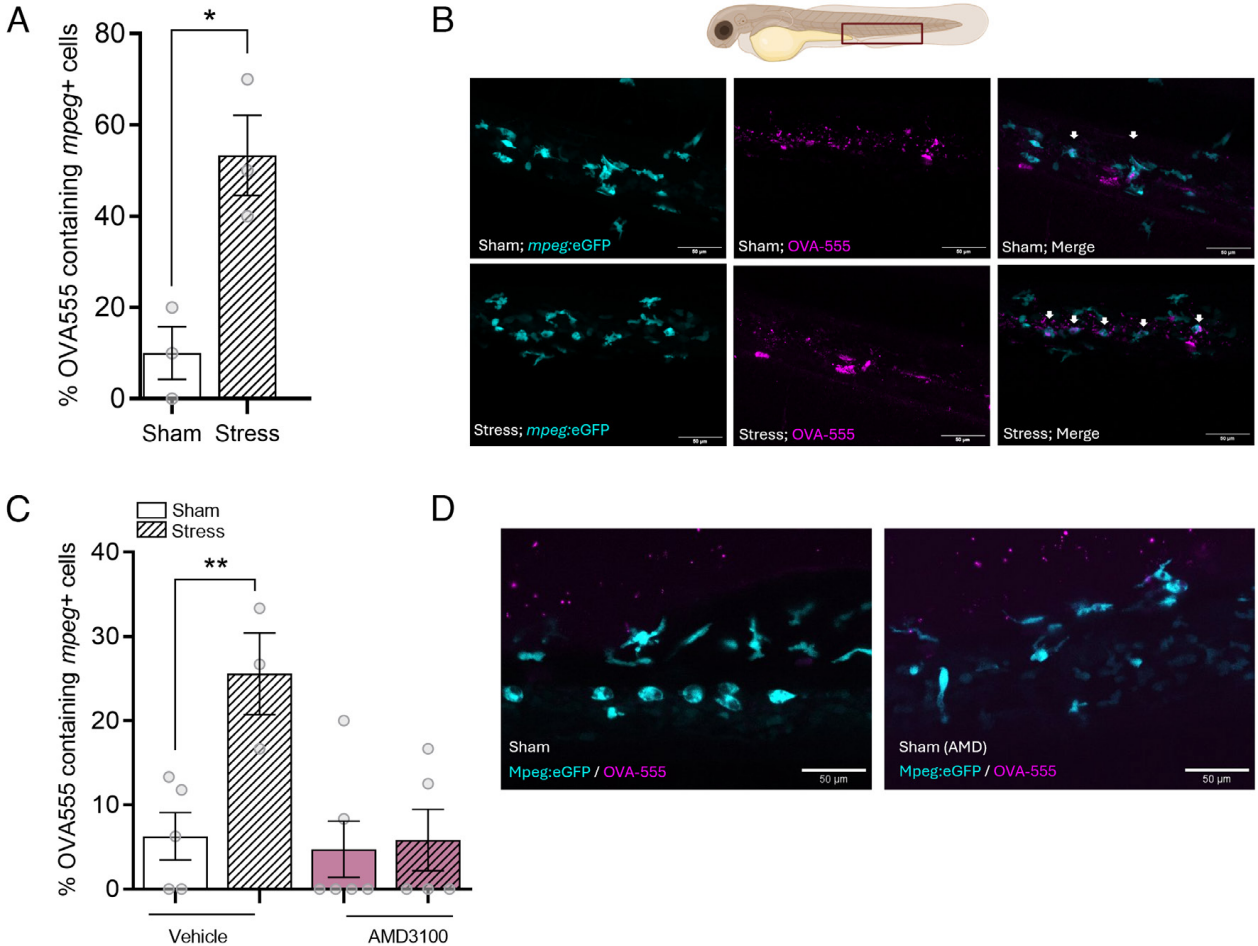
Acute stress increases the percentage of macrophage (*mpeg*+) that take up soluble antigens. (*A*) Percentage of macrophages that colocalize with the soluble antigen ovalbumin conjugated to the fluorescent dye Alexa555 (OVA-555) in sham and acutely stressed larvae. The number of colocalized events was normalized to the total number of macrophages in the CHT region. Data shown represent mean ± SEM, and dots represent measurements from single larvae. *P < 0.05. (*B*) Schematic diagram and representative fluorescence microscopy images of sham and acutely stressed *Tg(mpeg:eGFP)* larvae (4 dpf), treated with OVA-555 (shown in magenta). Arrows indicate *mpeg*+ macrophages that colocalize with the soluble antigen. (Scale bar, 50 µm.) (*C*) Percentage of macrophages that colocalize with OVA-555 in vehicle- and AMD3100-treated larvae. Data shown represent mean ± SEM, and dots represent measurements from single larvae. **P < 0.01. (*D*) Representative images of sham and acutely stressed *Tg(mpeg:eGFP)* larvae (4 dpf) upon vehicle or AMD3100 treatment, exposed to OVA-555 (magenta). Arrows indicate the *mpeg*+ macrophages that colocalize with the soluble antigen. (Scale bar, 50 µm.)

We next tested whether this subset of macrophages could increase barrier immunity by increasing the uptake of a soluble antigen. To accomplish this, we treated the fish with Alexa-555-conjugated ovalbumin (OVA-555) for 6 h and quantified the percentage of cells that were antigen-positive. In sham animals only ~10% of the observed macrophages accumulated OVA-555, but this increased remarkably to ~45% in the stressed animals (*P* = 0.0147; Fig. 5 *A* and *B*). To demonstrate that macrophages would first need to move to the DP to accumulate antigen, we treated larvae with AMD3100 to block the Cxcr4a-driven macrophage redistribution and studied the OVA-555 uptake. We observed that AMD3100-treated larvae showed no significant increase in the percentage of OVA-555-positive macrophages, whereas vehicle-treated larvae had a ~5-fold increase (Fig. 5 *C* and *D*). Therefore, our data suggest that the redistribution of macrophages upon acute stress is involved in antigen uptake from the environment and that acutely stressed animals will increase the macrophage population along barrier tissues to enhance immune surveillance in the skin.

## Discussion

In the present study, we demonstrate that acute stress causes the redistribution of leukocytes toward the skin of zebrafish larvae. In particular, the redistribution of macrophages is due to a highly specific mechanism by which cortisol increases macrophage *cxcr4a* expression by activating MR and GR. Macrophages will then localize to the skin, driven by an increased *cxcl12a* expression in melanocytes., We subsequently demonstrated that, due to this immune cell redistribution, acutely stressed zebrafish were better able to capture soluble antigens, providing immunological relevance to leukocyte migration poststress.

Available studies performed in mammals demonstrate that it is unlikely that the stress-induced immune enhancement observed in our study is restricted to fish. Our work builds on the seminal work of Dhabhar and McEwen, who demonstrated that in rodents, stress could be immune-enhancing and not just immune-suppressive (42). They described that acute stress results in an immune-enhancing phenotype with an increase in migrating leukocytes toward the skin in the absence or presence of an immune stimulus (10, 16). Here, we show that this response is well conserved within the vertebrate lineage, as zebrafish, the major model organism from the phylogenetic clade Actinopterygii (ray-finned fish), display a similar phenotype postacute stress. In other teleosts, invasion of leukocytes has been previously reported for many barrier tissues, such as the gill, intestine, or skin epithelium (43–45).

While we observed that both macrophages and neutrophils are redistributed poststress in our model, the skin-migratory response to acute stress is probably not limited to only innate immune cells, since Dhabhar et al. (46) demonstrated this effect for T cells and B cells. Other cells of the innate immune system, such as natural killer cells and monocytes have also been implicated as responsive to acute stress, and which cell-type predominates may depend on additional factors. For example, stress-induced enhancement of a skin-mediated immune response (DTH) was attributed to CD26L+ neutrophils, which did not increase postacute stress in IFN-γ receptor knockout mice. This is distinct from our macrophage response and suggests that neutrophils may only be recruited in response to skin inflammation. This is in line with our observation that the neutrophils only migrate to the DP in response to osmotic stress. When there is no additional inflammatory stimulus, the animal still mounts a robust immune response, which we postulate is preparatory in nature, corresponding to previous hypotheses that acute stress will act to prepare the animal for any subsequent challenges (42). It is worth noting that zebrafish larvae (<15 dpf) have not yet developed an adaptive immune response (25) and that neutrophils and macrophages are the main immune cells present <5 dpf. These cells are visualized using transgenic lines in which the expression of fluorescent proteins is driven by the *mpx*- or *mpeg1.1*-promoter, respectively, and are considered equivalent to their mammalian cellular orthologs, It remains unclear whether circulating monocyte precursors exist in zebrafish, although the *mpeg1.1* reporter line reveals some circulating forms and demonstrate their mobility throughout the animal (26). While adaptive immune cells may also be subject to stress-induced redistribution in zebrafish, this was not addressed in the present study.

Consistent with the results obtained in mice by Dhabhar et al. (10, 16), we also observed that the stress-induced increase in cortisol levels is a key driver of macrophage redistribution and that other stress factors, such as epinephrine, CRH, or ACTH, do not play a role. Building on this, we further identified that cortisol signaling through both MR and GR was required to elicit this effect. While it might be expected that increased cortisol levels during stress would primarily work through GR, we also show that MR activation is necessary to mediate macrophage redistribution, even though activation of MR would only require basal levels of cortisol. The idea that both MR and GR are required to mediate this response also touches on a broader subject within the nuclear receptor signaling field, whereby closely related nuclear receptors can heterodimerize through their conserved DNA binding domains (47, 48). Until recently there was little evidence that MR/GR heterodimers had any physiological relevance, although localization to specific response elements in the promoters of target genes had been demonstrated in the hippocampus poststress (49). In the present study we have revealed an MR/GR-driven phenotype in the immune system, but whether this involves heterodimerization remains to be determined.

When we determined the immunological targets of corticosteroid receptor signaling upon acute stress, we found that the expression level of a gene encoding the chemokine receptor Cxcr4 was increased. The *cxcr4* gene is a known target of glucocorticoid action in both fish (30) and mammals (50, 51). In zebrafish, there are two paralogues of this receptor, *cxcr4a* and *cxcr4b*, which have been characterized in both a developmental and immunological context (36). The *cxcr4a* expression was increased in response to the acute stressor, and this effect was dependent on the presence of a GRE within its promoter region. Future studies should explore whether the MR/GR dependency of stress-induced macrophage redistribution involves the binding of MR/GR heterodimers to this GRE. In contrast, the *cxcr4b* expression was not modulated upon stress, although the latter has previously been shown to be a target of glucocorticoid treatment in zebrafish macrophages (30). However, the increase in *cxcr4b* transcript abundance was only observed upon treatment with a glucocorticoid such as beclomethasone, suggesting that this gene is strictly GR- (and not MR-) responsive. The specificity of the *cxcr4a* induction upon acute stress is consistent with a model in which MR and GR together drive acute-stress-induced macrophage redistribution, while responses to chronic stress are primarily GR-mediated.

In mice, diurnal changes in corticosterone levels have been demonstrated to modulate Cxcr4 levels in lymphocytes, causing a redistribution of these cells from the blood to lymphoid organs such as lymph nodes, spleen, and Peyer’s patches, attracted by Cxcl12-producing cells (50). This supposedly allows for an enhanced immune response against infectious agents during the active phase of the diurnal cycle (50). It is interesting that these mouse lymphocytes show a similar Cxcr4/Cxcl12-based mechanism of action compared to the macrophages in our zebrafish model, suggesting that this signaling axis is a central mechanism of acute stress–induced immune enhancement, which is evolutionarily well conserved between vertebrates.

In addition, we demonstrate a clear role for melanocytes in recruiting immune cells to the skin poststress, which is in line with a well-established role for these cells as orchestrators of the innate immune response, especially in the context of cutaneous immune disorders (e.g., vitiligo). Classically, melanocytes were regarded solely as pigment-producing cells, but more recently, they have become recognized as important regulators of skin immunity (52). It is well established that melanocytes secrete immunoregulatory factors, including CXCL16 and CCL5 in response to interferon, CXCL8 in response to proinflammatory cytokines (i.e., IL-1, IL-6, TNF-α), and CXCL12 in response to lipopolysaccharide (LPS), attracting primarily lymphocytes, neutrophils, and macrophages, respectively (52–55). Taken together with our results, these findings make clear that the effect of acute stress on the innate immune system is specific, modulating the Cxcr4/Cxcl12 pathway to enhance macrophage recruitment toward the skin. In zebrafish, much like in humans, melanocytes are derived from neural crest cells and will migrate to various sites throughout the developing embryo, but are found primarily in the epidermis (56). In zebrafish embryos, melanocyte stem cells are located along the dorsal root ganglia, and mature melanocytes will migrate dorsolaterally and along nerves to form the embryonic patterns, which are distinct from the adult pigmentation pattern (56, 57). It will therefore be interesting to investigate in future studies whether melanocytes show a similar stress-induced immune-enhancing function in adult animals as well.

Our findings further establish a role for melanocytes, not only as key orchestrators of the innate immune response but also as key stress targets. The relationship between CNS-driven hormonal responses and the skin, also known as the skin–brain axis, has been well established. However, the majority of this research has been performed in the context of HPA axis-mediated changes in skin pigmentation (58). While stress-related hormones such as CRH and POMC-derived peptides like MSH and ACTH are known to have direct effects on the function of melanocytes (58), here we show that cortisol will directly act on melanocytes to increase the *cxcl12a* expression. In rainbow trout, it has been demonstrated that a single feeding of cortisol increased cytoplasmic extensions of melanocytes that penetrated into the basal layers of the epidermis and that at later time points, melanocyte extensions became apoptotic and were engulfed by macrophages. This supports that there is an important relationship between melanocytes and macrophages that can be mediated by cortisol (59). Since we found that cortisol increased the *cxcl12a* expression, it was surprising that, at least using established in silico techniques, no established GRE could be identified upstream of the *cxcl12a* promoter. This means that either cortisol, through MR and/or GR, does not directly regulate *cxcl12a* or that the response element is located more distally from the transcription start site.

The nature of melanocyte-derived CXCL12 is also intriguing from a human health perspective because this chemokine has the ability to not only recruit but also retain macrophages (60). Therefore, while stress-induced immune cell recruitment is enhancing under normal physiological conditions, it may become pathological if melanocytes retain macrophages or are unable to properly regulate Cxcl12 expression levels. Indeed, stress is known to exacerbate several inflammatory skin disorders (61), but little is known about the role of either Cxcl12 or melanocytes in these conditions. Overall, melanocyte-mediated immune cell recruitment is not only a key aspect in response to acute stress but also may represent a key player in various skin-related disorders.

While it has often been postulated that the role for enhanced migration of leukocytes toward barrier tissues poststress would be to increase immune defense strategies, the exact role that these cells play in barrier immunity under stressful conditions has been largely unexplored. In the present study, we show that the redistributed subset of macrophages is involved in increased antigen uptake from the environment, thereby playing a role in enhanced immune surveillance after stress. The role of the redistributed macrophages in the skin could look different when studying aquatic versus terrestrial vertebrates. While for zebrafish macrophages the aquatic environment of the skin allows for sampling soluble antigens from the water, such antigen sampling may be limited in terrestrial vertebrates to mucosa-covered barrier tissues, such as the gut or lungs. In other tissues, such as the skin, the role of macrophages would likely be almost entirely preparatory. A recently identified ectoderm-derived myeloid-like cell population has also been suggested to play a role in antigen uptake from the environment in zebrafish. These cells were named metaphocytes and are also *mpeg*-positive and *mfap4*-negative (40). However, our data showed no increase in the metaphocyte-specific markers *spic* and *grn2* after stress, which is expected because metaphocytes do not start to develop until 4 dpf (39). Alternatively, these cells could be Langerhans-like cells, which represent the tissue resident macrophage population in the skin (62). However, this option is unlikely since Langerhans cells are known to express *mfap4* (40, 41). Therefore, the exact identity of the redistributed macrophage subpopulation, that is *mpeg*-positive and *mfap4*-negative, remains to be established.

In summary, here we show that immune cells, in particular macrophages, migrate toward the skin in response to acute exposure to stress. This cortisol-mediated phenomenon that leukocytes migrate toward the barrier tissues (i.e., the skin) has been shown before in rodents, indicating that this stress-induced immune enhancement is conserved across vertebrate taxa. Despite the previous description of this phenotype, there is a dearth of information regarding the molecular mechanisms, which has largely precluded any therapeutic advantages this process may afford. We demonstrate that macrophages increase Cxcr4 expression upon stress in an MR- and GR-dependent way, thereby facilitating chemotaxis toward Cxcl12-expressing melanocytes. This has previously been shown for T-cells, pointing to a central mechanism of action of stress-induced immune enhancement. Finally, we establish the immunological significance of these stress-induced redistribution events, as they appear to be involved in immune surveillance in the skin and thereby increase barrier immunity. Together, these findings highlight a conserved mechanism through which acute stress enhances innate immune function via corticosteroid-driven leukocyte redistribution, enhancing our understanding of the molecular pathways linking stress and immunity.

## Materials and Methods

### Zebrafish Husbandry and Handling.

Zebrafish were maintained in accordance with guidelines from the Zebrafish model organism database (zfin.org), the EU Animal Protection Directive 2010/63/EU, and in compliance with the directives of the animal welfare body at Leiden University (License No. 10612). All experiments were performed on larval stages (before the free feeding stage and before sexual differentiation), which do fall under animal experimentation under EU legislation. Briefly, fish were held in a recirculating system on a 14 h:10 h light:dark cycle (light on 08:00 h; light off 22:00 h). Water was maintained at 28 °C, 300 μS conductivity, and a pH of 7.5. Fish were fed twice daily, consisting of Gemma Micro 500 (Skretting, Stavanger, Norway) in the morning and live artemia in the afternoon. Fertilization was performed by natural spawning at the beginning of the light period. Eggs were collected, and embryos and larvae were reared for days 0 to 5 dpf in a 28.5 °C incubator in 10 cm Petri dishes (Sarstedt, Nümbrecht, Germany) at a density of 100 embryos/dish in E3 embryo media (5 mM NaCl, 0.17 mM KCl, 0.33 mM CaCl_2_, 0.33 mM MgSO_4_ + 0.1 ppm methylene blue antifungal agent). Embryos and larvae were raised on a 14 h:10 h light: dark cycle, and 50% of the embryo media was refreshed daily.

### Zebrafish Lines.

The following lines were used in this work: *Tg(mpx:GFP)i114; Tg(mpeg1.1:mCherry-F)ump2*, *Tg(mpeg1.1:eGFP)gl22; Tg(mfap4:mCherry-F)ump6*, Casper mutants (*mitfa^w2^*/*mpv17^a9^*), the *cxcr3.2* mutant strain *Tg(mpeg1.1:mCherry-F)ump2;cxcr3.2^hu6044^* and the associated wild-type siblings *Tg(mpeg1.1:mCherry-F)ump2;cxcr3.2^+/+^.*

### Pharmacological Treatment.

To determine whether cortisol was involved in stress-induced leukocyte distribution, embryos were treated with metyrapone (100 µM; Sigma-Aldrich, St. Louis, MO), an 11β-hydroxylase inhibitor, 16 h prior to the stressor. To assess corticosteroid receptor involvement, embryos were treated with an appropriate antagonist GR: mifepristone (1.25 µM; Sigma-Aldrich); MR: eplerenone (1.25 µM; Sigma-Aldrich) 16 h prior to the stressor or cortisol. To assess whether MR and/or GR activation was required to facilitate macrophage redistribution, we treated larvae for 1 h with either a GR agonist, beclomethasone (25 µM; Sigma-Aldrich), dexamethasone (10, 25 µM; Sigma-Aldrich), or prednisolone (25 µM; Sigma-Aldrich), an MR agonist, aldosterone (10 µM; Sigma-Aldrich), or a GR/MR agonist cortisol (hydrocortisone; 25 µM; Sigma-Aldrich). Posttreatment larvae were anesthetized in MS222 (0.168 mg/mL) and washed 3× in E3 media. Larvae were then allowed to recover for 24 h, after which macrophage redistribution was quantified as described below. To determine the involvement of Cxcr4, we treated fish with AMD3100 (35 µM), which has been previously used in zebrafish (37).

### Larval Stress.

Zebrafish larvae were stressed as described previously (63, 64). Briefly, at 12.00 h, larvae were quickly transferred to 50 mL Falcon tubes. Each tube contained a final volume of 20 mL of embryo media. For determining the cortisol response, the time 0 larvae (unstressed larvae) were immediately killed (MS222, Millipore-Sigma, final concentration 0.4 mg/mL). The remaining larvae were placed on an orbital shaker and subjected to a vortex stressor (250 rpm) for 2 min, allowed to recover, and sampled [each sample measured (n) is a pool of 10 larvae] at the appropriate time points (5, 10, or 30 min). To assess the role of acute stress on macrophage movement and function, fish were stressed as described above and allowed to recover in petri-dishes in a 28 °C incubator for 24 h. Additional information regarding osmotic and light stressors can be found in *SI Appendix*.

### Cortisol ELISA.

Cortisol levels were measured as described earlier for zebrafish (31).

### Quantitative Real-Time PCR.

Transcript levels of specific genes were measured by quantitative real-time PCR (qPCR). Total RNA was extracted from larvae using Trizol reagent (Thermo Fisher Scientific, Waltham, MA) according to the manufacturer’s instructions and quantified using a Nanodrop (Thermo Fisher Scientific). RNA (1 mg) was treated with DNase I (Thermo Fisher Scientific) using the High-Capacity cDNA Reverse Transcription Kit (Thermo Fisher Scientific), according to the manufacturer’s protocols. Transcript levels were measured by qPCR using SYBR green (Sso Advanced; Bio-Rad, Hercules, CA) in duplicate using gene-specific primers as described previously (30) (*SI Appendix*, Table S1).

### Live Imaging.

Imaging using a stereo fluorescence microscope was done by placing zebrafish larvae (4 dpf) on a petri dish with 2% agarose. Larvae were anesthetized with 0.168 g/L buffered MS222 (2:1 sodium bicarbonate; Sigma-Aldrich). The fluorescently labeled macrophages and neutrophils were visualized using a Leica MZ16FA fluorescence stereomicroscope supported by LAS 3.7 software (Leica Microsystems, Wetzlar, Germany). The numbers of macrophages and neutrophils were quantified by manual counting after blinding of the samples.

Confocal imaging was done as previously described (30). Briefly, larvae were mounted in 0.7% low melting agarose containing 0.2 mg/mL MS-222 (Sigma-Aldrich) as a sedative/anesthetic in E3 medium. Confocal imaging was performed using a Leica Stellaris 5 (Leica Microsystems). Imaging of the GFP signal was performed using the 488 nm laser, and imaging of the mCherry signal was performed using the 561 nm laser. Analysis of imaging data was performed using ImageJ (FIJI).

### CRISPR/Cas9 Mutagenesis: Generation of *mitfa* Crispant (F0) Larvae.

To disrupt the production of melanocytes in the *Tg(mpx:GFP/mpeg1.1:mCherry-F)i114/ump2*, we injected three guides targeting the *mitfa* gene, as previously described in zebrafish (65). Ribonucleoprotein (RNP) complexes (guide RNA+ Cas9 protein) were generated as previously described (65). Briefly, CRISPR RNA (crRNA) for the regions of interest (mitfa_cr1: CACGGCATGACCCCGGGACC; mitfa_cr2: AAGCCTGCCGGCCGTCAAAA; mitfa_cr3: ATGGACAAAGCTGGACCATG) was designed using ChopChop (https://chopchop.cbu.uib.no/), and guide RNA (gRNA) was generated by annealing the specific crRNA and universal tracrRNA (both from IDT, Leuven, Belgium) at 95 °C (5 min). RNPs were assembled by hybridizing the gRNA to the Cas9 protein (IDT) at 37 °C (5 min). RNPs were microinjected into fertilized embryos prior to the single-cell stage. To disrupt the production of melanin, guides targeting the gene for tyrosinase (tyr_cr1: AGTGCGCCGGAAACTACATG; tyr_cr2: ACGACCGGATCCGGGTACCG; tyr_cr3: TCCGTCGTTGTGTCCGATGG) were assembled into RNPs, as described above, and injected into fertilized embryos prior to the single cell stage. In both cases, a lack of pigment was used to screen larvae for a successful knockdown.

### CRISPR/Cas9 Mutagenesis: GRE Mutation.

To identify GREs in the proximity of the *cxcr4a* transcriptional start site, we used the JASPAR TFBS2022 track of the University of Santa Cruz (USCS) genome browser (genome build: GRCz11/danRer11). There was a single putative GRE upstream of the *cxcr4* gene (chr6:12921929-12921945; *P* > 0.0001; aggacgtcgtcttct) located approximately 227 bp upstream of the transcriptional start site. To mutate the identified GRE, ribonucleoprotein (RNP) complexes (guide RNA+ Cas9 protein) were generated as previously described above. The CRISPR RNA (crRNA) for the region of interest (*cxcr4a_crRNA*: GAAGAAGACGACGTCCTGTGGGG) was assembled into an RNP and microinjected into fertilized embryos, prior to the single-cell stage. To identify indels we genotyped by fragment analysis as previously described (65) (*Forward genotyping primer:* GGATGTTGTGTTTGATAGGCAA; *Reverse genotyping primer:* GCGCTCGAACAGTTTAAATACC) Briefly, the area of interest was amplified by quantitative PCR (BioRad CFX96 with cycling conditions, 95 °C (3 min), 40 cycles of 95 °C (30 s), 60 °C (30 s), 72 °C (30 s), followed by a 5 min extension at 72 °C) after which a melt curve was run to ensure a single amplicon, where a shift in the amplicon size was seen as a change in amplicon size due to the presence of indels.

### Antigen Uptake Study.

To determine the immunological significance of stress-induced macrophage redistribution, we assessed the amount of antigen uptake, as described previously in zebrafish (39). Briefly, larvae, either sham or 24 h poststress, were immersed in 2.5 µg/mL Alexa Fluor 555 conjugated ovalbumin (OVA-555; Invitrogen, Waltham, MA), in a 12-well dish. Larvae were exposed to OVA-555 for 6 h. Live zebrafish imaging was done from 4 to 6 h postexposure.

### Quantification and Statistical Analysis.

Statistical analyses were performed using GraphPad Prism 6. Data were log-transformed where necessary to meet the assumptions of normality and equal variance. Untransformed data are shown in all graphs.

#### qPCR.

Transcript abundance was analyzed using the delta–delta CT method. Results are shown as mean ± SEM (^ns^*P* > 0.05, *P % 0.05, **P % 0.01, ***P % 0.001). A two-tailed *t* test was used to determine significant differences between mRNA levels of chemokine receptors. For cxcr4GRE experimental results, a two-way ANOVA was performed.

#### Assessment of macrophage distribution.

To quantify the percentage of macrophages that redistributed to the DP, the number of macrophages within 10 μm along the dorsal edge of the fish (excluding the fin fold) was divided by the total number of macrophages in the tail region (determined by the area directly posterior to the yolk). Results are shown as mean ± SEM (^ns^*P* > 0.05, *P % 0.05, **P % 0.01, ***P % 0.001). A two-way ANOVA was used to determine differences in leukocyte distribution over time, post–metyrapone treatment, post-AMD 3100 treatment, post-cxcr4aGRE mutagenesis, and studies involved abolishment of melanocytes. A one-way ANOVA was used to determine significant differences in the number of macrophages in response to cortisol and beclomethasone, while the *t* test was used to determine significant differences between mRNA levels of chemokine receptors.

#### Determination of antigen uptake.

To quantify the number of cells that were positive for antigen uptake, three independent larvae were imaged. The amount of uptake in macrophages of the CHT region were quantified by dividing the number of cells with antigen signal by the total number of cells in the region. Results are shown as mean ± SEM (^ns^*P* > 0.05, *P % 0.05, **P % 0.01, ***P % 0.001). A two-tailed *t* test was used to determine significant differences between sham versus stressed fish, while a two-way ANOVA was used to determine significant differences post-AMD3100 treatment.

## Supplementary Material

Appendix 01 (PDF)

Movie S1. Video showing the localization and migration at the dorsal periphery of macrophages (shown in red), in *Tg(mpeg1.1:mCherry/mpx:GFP/kdrl:mTurqoise)* zebrafish larvae (3 dpf). Neutrophils are shown in green, the vasculature is shown in blue. Larvae were imaged from 1h until 24h post stress.

## Data Availability

All study data are included in the article and/or supporting information.
