# Supplementary material for: Melanocyte-dependent macrophage redistribution enhances skin immunity upon acute stress
Source: Proc Natl Acad Sci U S A. 2025 Aug 19;122(34):e2511358122. doi: 10.1073/pnas.2511358122 (PMC12403131; doi:10.1073/pnas.2511358122)
Supplement: Supplementary file 1 — Appendix 01 (PDF) [file pnas.2511358122.sapp.pdf]

SUPPLEMENTAL INFORMATION

Melanocyte-dependent macrophage redistribution enhances skin immunity upon acute stress

Erin Faught*, and Marcel JM Schaaf*

Institute of Biology Leiden, Leiden University, Leiden, The Netherlands

Supplemental Video 1. Video showing the localization and migration at the dorsal periphery of macrophages (shown in red), in *Tg(mpeg1.1:mCherry/mpx:GFP/kdrl:mTurqoise)* zebrafish larvae (3 dpf). Neutrophils are shown in green, the vasculature is shown in blue. Larvae were imaged from 1h until 24h post stress.

Supplemental Table 1:

Gene	Forward Primer (5'-3')	Reverse Primer (5'-3')	Source
ccr2	TGGCAACGCAAAGGCTTTCAGTGA	AGGTTTCCCGAAGGTGAAGT	Xie *et al*., 2019[1]
ccl2	GTCTGGTGCTCTTCGCTTTC	TGCAGAGAAGATGCGTCGTA	Xie *et al*., 2019[1]
cxcr4a	CATGACAGACAAGTACCGTCT	TGCTGTACAAGTTTACCGTGTA	Peng *et al*., 2022[2]
cxcr4b	TGCTAACATTCCTGATAAGACC	GTACTTTTATTGCCAGACCTAAAGG	Peng *et al*., 2022[2]
cxcl12a	GCAAGTGCTTTGACACAAAAAG	TTTGTTTGGCAAAGTAACCCTG	Peng *et al*., 2022[2]
cxcl12a	GATCGTGATAGCTTTGTGAACC	AATGTTAACAATGCTTGGCCTC	Peng *et al*., 2022[2]
fkbp5	CGAGCAGGAGATGGGAACC	CAACGGAAACGCTCATTGC	Chatzopoulou *et al*., 2017[3]
actb1	CGAGCAGGAGATGGGAACC	CAACGGAAACGCTCATTGC	Chatzopoulou *et al*., 2017[3]

1. Dis Model Mech 12. 10.1242/dmm.037887
2. Elife 12. 10.7554/eLife.90048
3. Mol Cell Endocrinol 447, 61-70. 10.1016/j.mce.2017.02.036

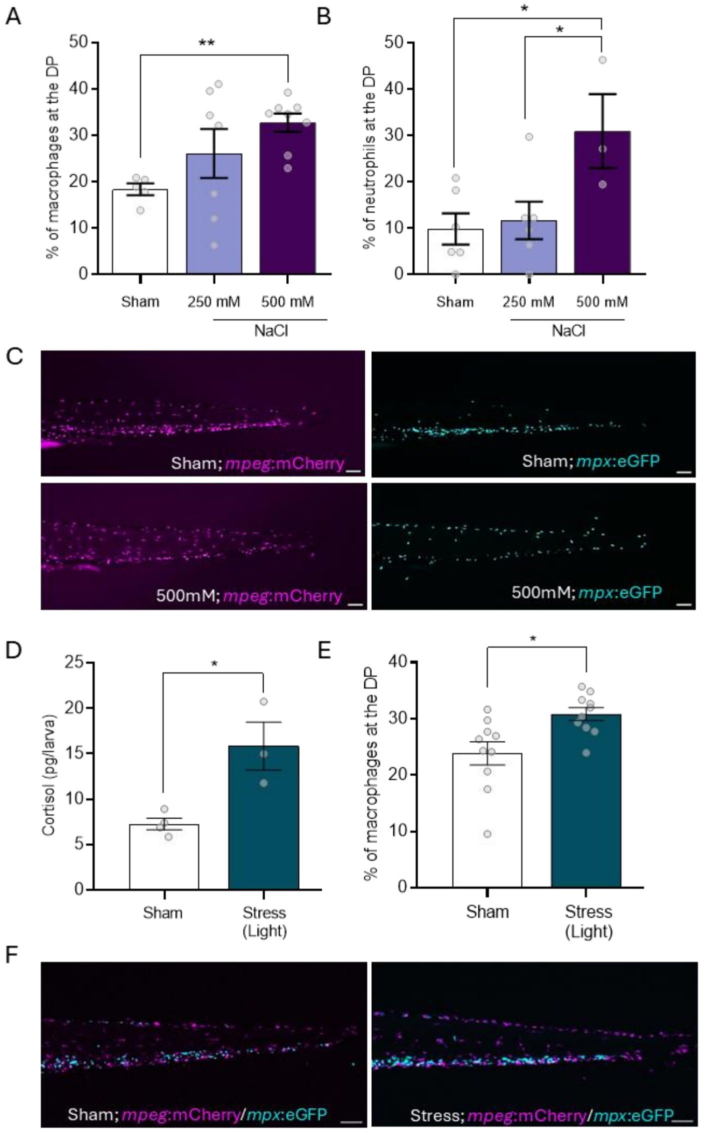

Figure S1. Acute Osmotic and Light Stress induce leukocyte distribution towards the dorsal periphery. A,B. Percentage of macrophages (*mpeg*+; shown in A) or neutrophils (*mpx*+; shown in B) localized at the dorsal periphery (DP) in control larvae, or larvae treated with half strength sea water (250 mM) or full strength sea water (500 mM) for 5 min. A sham group that was washed similarly to the treated larvae was also included. **C.** Representative fluorescence microscopy images of the tail region of *Tg(mpeg:mCherry/mpx:eGFP)* larvae after osmotic stress. Scale bar: 100 µm. **D**. Cortisol levels in zebrafish larvae subjected to a light stressor (15 min dark acclimation, followed by 10 min white light, adapted from De Marco et al., 2013[1]). **E.** Percentage of macrophages (*mpeg*+) localized at the dorsal periphery (DP) in sham or light-stressed larvae. **F**. Representative fluorescence microscopy images of the tail region of *Tg(mpeg:mCherry/mpx:eGFP)* sham or light-stressed larvae. Scale bar: 100 µm. Data shown represent mean ± SEM, and dots represent measurements from single larvae. *p<0.05, **p<0.01.

1. *Front Neural Circuits 7:82. 10.3389/fncir.2013.00082*

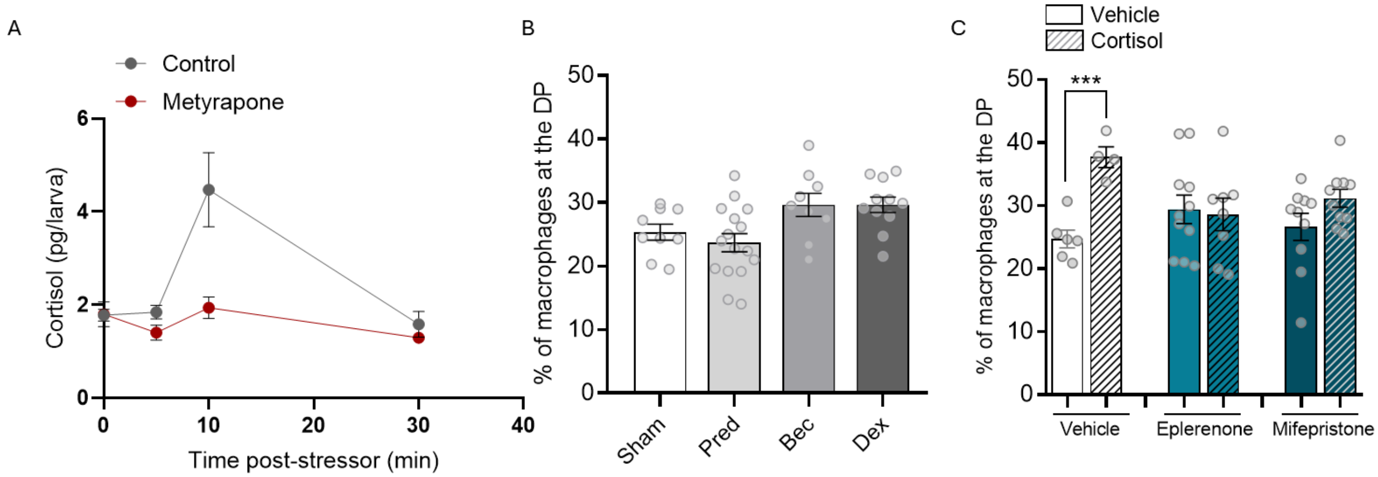

Figure S2. Metyrapone abolishes the stress-induced cortisol increase. A. Whole body cortisol levels, determined by ELISA, of 3 dpf larvae that have been subjected to acute stress (swirling (250 rpm; 2 min)), treated with either vehicle (0.05% ethanol), (100 μM; 16h). **B.** Percentage of macrophages that redistribute to the dorsal periphery (DP) 24h post-acute glucocorticoid treatment (1h). *Tg(mpeg:mCherry)* larvae were treated for 1h with either vehicle (0.05% ethanol), prednisolone (25 μM; 1h), beclomethasone (25 μM; 1h), or dexamethasone (25 μM; 1h). **C.** Percentage of macrophages that migrate towards the DP post acute cortisol treatment (25 μM; 1h) in *Tg(mpeg:mCherry)* larvae previously treated with either vehicle (0.05% ethanol), eplerenone (MR antagonist; 1.25 μM; 16h) or mifepristone (GR antagonist; 1.25 μM; 16h). Data shown represent mean ± SEM, and dots represent measurements from single larvae. *p<0.05, ***p<0.001.

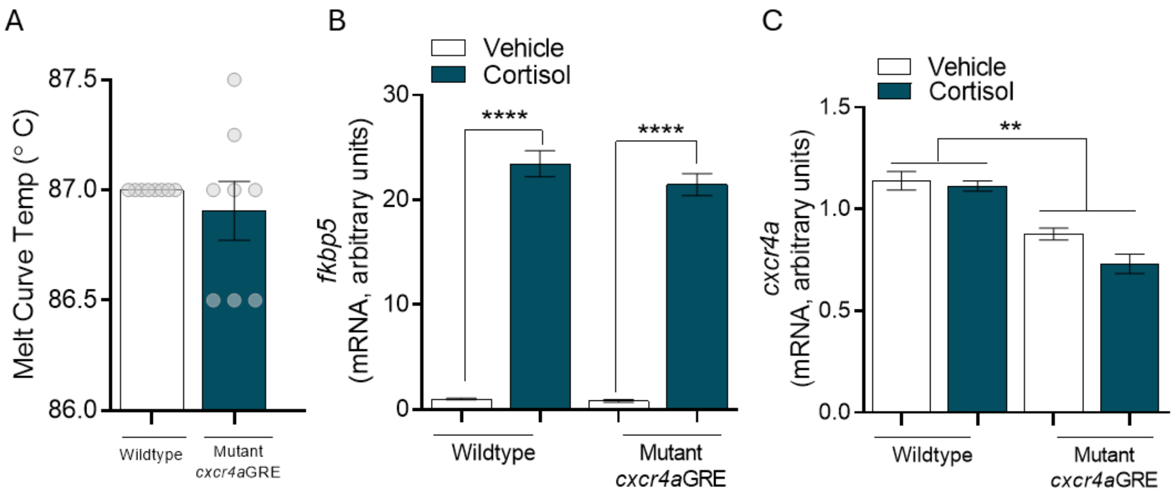

Figure S3. Disruption of the putative GRE in the proximal promoter of the *cxcr4a* gene using CRISPR/Cas9-mediated gene editing. A. Amplicon size, as determined by melt curve temperature, in wildtype and crispant larvae with a disruption of a GRE in the promoter of *cxcr4a*. **B.** Transcript abundance of *fkbp5* in in wildtype and *cxcr4a* GRE injected larvae treated with cortisol (5 µg/ml) for 6 h. **C.** Transcript abundance of *fkbp5* in in wildtype and *cxcr4a* GRE injected larvae treated with cortisol (5 µg/ml) for 6h. In all graphs, data shown represent mean ± SEM, and dots represent individual measurements (single larvae in A, pools of 10 larvae in B and C). *p<0.05, **p<0.01.

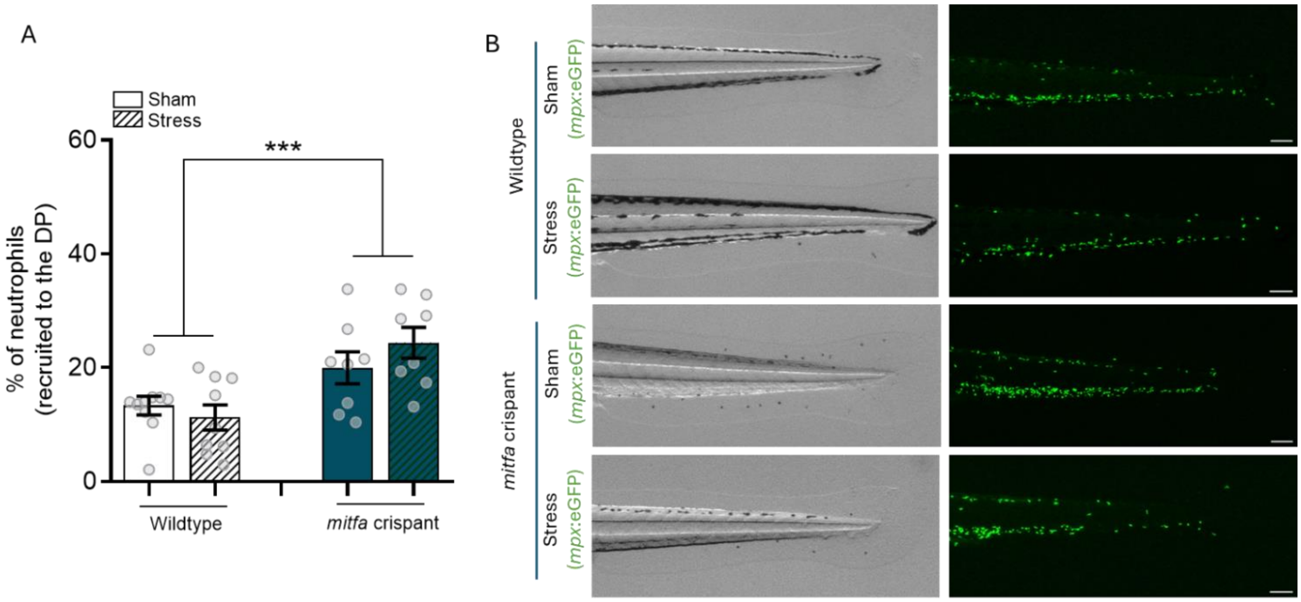

Figure S4. Neutrophil distribution is disrupted in *mitfa* crispant larvae. A. Percentage of neutrophils (*mpx*+ cells) that migrate towards the dorsal periphery in wildtype or *mitfa* crispant larvae subjected to sham treatment or acute stress. Data shown represent mean ± SEM, and dots represent measuremnts from single larvae. ***p<0.001. **B.** Representative brightfield and fluorescence microscopy images of the tail region of wildtype and *mitfa* crispant *Tg(mpeg:mCherry/mpx:eGFP)* larvae, subjected to sham treatment or acute stress. Scale bar: 100 µm.

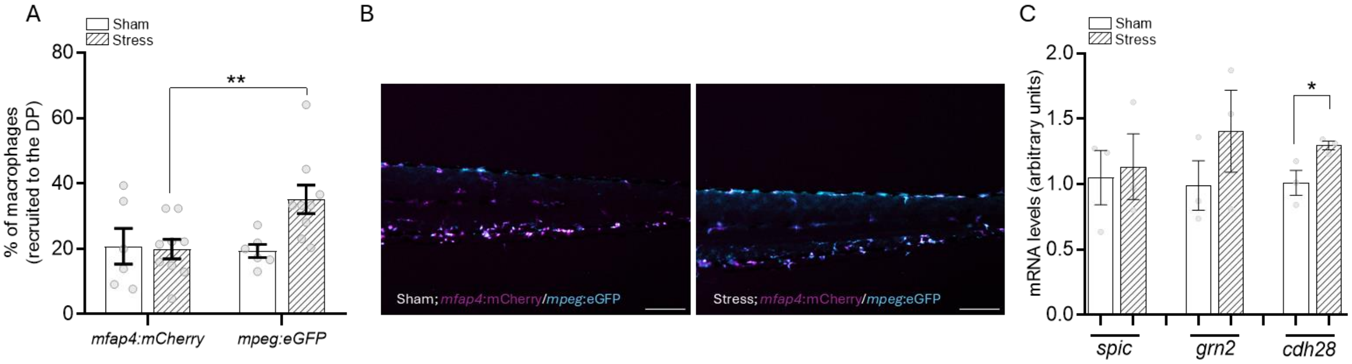

Figure S5. Macrophage distribution is disrupted in *mitfa* crispant larvae. A. Percentage of macrophages (*mpeg*+ or *mfap4*+) that migrate towards the dorsal periphery (DP) 24h post acute stress in *Tg(mfap4:mCherry/mpeg:eGFP)* larvae. Data shown represent mean ± SEM, and dots represent measurements from single larvae. $^{**}p<0.01$. **B.** Representative fluorescence microscopy images of sham-treated and stressed *Tg(mfap4:mCherry/mpeg:eGFP)* larvae (4 dpf). Scale bar: 100 μm. **C.** Transcript abundance of genes (*spic*, *grn2,* and *cdh28)* which are known to be expressed in macrophage-like cells (metaphocytes), in sham-treated and stressed wildtype larvae (4 dpf). Data shown represent mean ± SEM, and dots represent measurements from pool of 10 larvae. $^{*}p<0.05$.
